# What is the value of collecting detailed costing data in clinical trials?

**DOI:** 10.1186/1745-6215-12-S1-A42

**Published:** 2011-12-13

**Authors:** Helen Dakin, Giselle Abangma, Sarah Wordsworth

**Affiliations:** 1 Health Economics Research Centre, University of Oxford, Oxford, OX3 7LF, UK

## Objectives

Cost data for trial-based economic evaluation can be obtained through micro-costing (collecting resource use and unit cost data for each centre or patient), gross-costing (average costs based on top-line budgets) or provider tariffs (e.g. healthcare resource groups, HRGs). Most studies use a combination of approaches due to data availability, although there is little guidance on which is best. We report a systematic comparison of the three costing approaches in IVAN: a non-inferiority randomised controlled factorial trial of treatment regimens for age-related macular degeneration (AMD), where policy makers are interested in the efficacy and cost-effectiveness of two dosing regimens of bevacizumab (Avastin) and ranibizumab (Lucentis). We aimed to assess the extent to which micro-costing, gross-costing and HRGs differ, and to investigate resource use variation between UK hospitals and explore possible reasons for this variability.

## Methods

We compared micro-costing, gross-costing and HRG estimates of consultation costs using IVAN data. Nineteen IVAN trial centres were sent questionnaires on the resources required to set up and run clinics. Resources were valued using national unit costs to give micro-costing estimates that are compared against Department of Health gross-costing estimates and the HRG for ophthalmology outpatient consultations. Regression analyses explore the variability between centres.

## Results

Fourteen centres (74%) returned questionnaires. The mean cost of a follow-up ophthalmology outpatient consultation is £74 compared with an HRG cost of £53. Preliminary micro-costing suggests that both HRGs and gross-costs substantially underestimate the cost of consultations to administer treatment (excluding drug costs) or monitor outcomes. Micro-costing also highlighted substantial variation in consultation costs, facilities, organisation and resource use not captured within HRGs or gross-costs. Clinic size did not explain variations in consultation costs.

## Conclusions

Although data analysis is ongoing, initial results suggest that micro-costing estimates for administration and monitoring of Avastin/Lucentis are higher than gross-costs or HRGs. HRG costs were lowest, suggesting that hospitals must cut costs substantially to break even on such consultations. Differences in costing methodology are likely to affect cost-effectiveness results: particularly in the context of a non-inferiority trial comparing different dosing regimens, where cost differences will drive conclusions about cost-effectiveness. Only micro-costing differentiated between consultations for monitoring and drug administration. Micro-costing (unlike other approaches) also showed how costs and patient management vary between UK centres, facilitating analysis of heterogeneity and identification of potential efficiency improvements. This demonstrates the value of collecting detailed resource use data in trials.

